# Association between Hand Digit Ratio (2D : 4D) and Acute Lymphoblastic Leukemia

**DOI:** 10.1155/2018/4938725

**Published:** 2018-11-26

**Authors:** Verônica O. Dias, Maria L. Santos, Patrícia Helena C. Mendes, Claudia A. D. Fonseca, Daniella R. B. Martelli, Rodrigo S. de Andrade, Hercílio Martelli Júnior

**Affiliations:** ^1^Health Science Program, State University of Montes Claros, Montes Claros, Minas Gerais, Brazil; ^2^Dental School, State University of Montes Claros, Montes Claros, Minas Gerais, Brazil; ^3^Medicine School, State University of Montes Claros, Montes Claros, Minas Gerais, Brazil; ^4^Dental School, State University of Campinas, Piracicaba, São Paulo, Brazil

## Abstract

**Objective:**

Digit ratio (2D : 4D) has been suggested as a biomarker for prenatal hormone activity and has been linked to several types of cancer. This study investigated the possible correlation between 2D : 4D ratios and acute lymphoblastic leukemia.

**Methods:**

A case-control study was performed with Brazilian subjects. Direct measurements of the lengths of index and ring fingers of both hands of patients with acute lymphoblastic leukemia (*n* = 43) and controls matched by age and gender (*n* = 86) were obtained by using a digital vernier caliper. Mean ratios between the second and fourth digits were compared. Data were analyzed by Student's *t*-test with a significance level of 5%.

**Results:**

No significant difference was found between the mean digit ratios of the right and left hands between the groups for any analysis (*p* > 0.05), neither for the whole sample nor for the distribution by gender.

**Conclusions:**

We observed that patients with acute lymphoblastic leukemia do not have a different digit pattern when compared with unaffected individuals, which may suggest that exposure to prenatal sex hormone is similar between groups.

## 1. Introduction

The ratio of the length of the second (index) finger to the fourth (ring) finger (also known as digit ratio or the 2D : 4D ratio) has been proposed as a marker for prenatal hormone (testosterone and estrogen) exposure [1]. Digit ratio is a sexually dimorphic trait, being constant since birth and not influenced by the adult hormone levels [2]. A high 2D : 4D ratio means higher estrogen exposure, and a low 2D : 4D ratio suggests higher testosterone exposure [3].

Evidence also set out that 2D : 4D may be predictive of susceptibility to some types of cancers, and that this may be particularly true for cancers that show sex differences in their occurrence, progression, and/or prognosis [4–6]. Digit development is regulated by the activity of 19 skeletogenic genes with estrogen and testosterone regulating their expression in the opposite directions [4, 7]. Importantly, some of these genes were also strongly implicated in the development and progression of cancer [4].

Testosterone and progesterone have been shown to regulate *HOX* gene expression [8]. *HOX* gene regulates essential aspects of embryogenesis, morphogenesis, and cell differentiation of different areas of the human body and has been shown to be deregulated in acute leukemia in childhood [9, 10]. Therefore, if 2D : 4D has been proposed as a marker for prenatal hormone (testosterone and estrogen) exposure, it could also be linked to leukemia subtypes in childhood since hormonal exposure in early life has been associated with risk of testicular cancer, breast cancer, and childhood leukemia [5, 11, 12].

Acute lymphoblastic leukemia (ALL) is the most common neoplasm in children under 5 years old, accounting for about 80% of leukemia cases in this age group and more frequently in boys than in girls (male : female, 55% to 45%) [13]. In Brazil, the incidence of ALL is about 40 cases per million people younger than 15 years of age, with the peak incidence occurring at 2 to 5 years of age [14]. Several genetic factors are associated with an increased risk of ALL, but most patients have no recognized inherited factors. Increased rates of the disease have been linked to exposure to radiation and certain chemicals, but these associations explain only a minority of cases [13].

The 2D : 4D and ALL have been associated with prenatal hormonal exposure. Therefore, we hypothesized that 2D : 4D may be a potential marker for ALL. We aimed to investigate whether individuals with ALL have a different standard digit ratio when compared with a similar unaffected population. To our knowledge, there are no studies reporting the potential relationship between 2D : 4D and ALL.

## 2. Methods

### 2.1. Samples

All patients with acute lymphoblastic leukemia (ALL group) from February 2016 to September 2017, from the reference hospital in oncology, in the town of Montes Claros, Minas Gerais State, Brazil, were evaluated. The following inclusion criteria were applied: (1) having a diagnosis of ALL, (2) undergoing treatment at the institution, and (3) age between 2 and 17 years.

After identifying the cases (*n* = 43), a control group (*n* = 86) was selected among patients who were assisted by primary-care physicians in health public services and in the University General Hospital. The controls were children free from cancer, with age and sex matched to the case group. Exclusion criteria of both groups included history of fractures on the fingers of hand, hormonal disorders, and presenting clinical syndromes resulting from chromosomal abnormalities, such as Down syndrome (data reported by parents or guardians and medical records).

All subjects enrolled in this study reside in the same geographical area (northern part of Minas Gerais State, Brazil). Thus, the control group presented similar demographic, ethnic, and sociocultural characteristics to the ALL group. Written informed consents were obtained, and the study was carried out with the approval of the University Human Research Ethics Committee (1.416.786/2016).

### 2.2. 2D : 4D Measurements

The lengths of the second (index) and fourth (ring) fingers were measured using digital vernier calipers with a resolution of 0.01 mm. Two measurements were taken, both by the same researcher; the second measurement was blind to the first and was performed 30 minutes after the first one. Intraobserver reliability was high for digit measurements, with intraclass correlation coefficients (ICC) for 2D = 0.995, 4D = 0.998, and 2D : 4D ratio = 0.993 [15]. Measurements were taken from the tip of the finger to the basal crease. When two creases were visible at the base of the digit, the crease proximal to the palm was chosen. The analyzed ratio was the mean of the two measurements performed. Measurements were taken on both hands, and the right minus left 2D : 4D was calculated. We identified them as right-hand digit ratio (R2D : 4D) and left-hand digit ratio (L2D : 4D). The difference between the right and left 2D : 4D is known as DR-L [16].

### 2.3. Statistical Analysis

A descriptive and comparative statistical analysis was initially carried out with baseline characteristics from both groups, such as gestational age (week), birth weight (g), birth order, mother's age at child's birth (year), mother's educational level, and family history of ALL (first degree relatives). This analysis was performed using a chi-squared test. Cancer is a multifactorial disease in which both genetic and environmental factors could play significant roles [13], and since the etiology of ALL remains unknown, it is important to evaluate the baseline characteristics of both groups in the control-case study groups.

Student's *t*-test for unpaired samples was performed to compare the means of 2D : 4D ratios and DR-L among groups. The effect size, which consists of a measure of standardized magnitude that represents the importance of association in practical terms, was also calculated, measured by Cohen's d [17]. All analyses were conducted using SPSS® 23.0 for Windows (IBM SPSS, Armonk, NY USA) with a significance level of 5%.

## 3. Results

The age of the individuals in both groups ranged from 3 to 17 years with a mean age for the ALL group of 9.02 years (±4.1) and for the control group of 7.74 years (±2.79). The proportion of boys and girls for the ALL group was 55.8% and 44.2%, respectively, and the proportion of boys and girls for the control group was 50.0% and 50.0%, respectively (*p* = 0.53).

Baseline characteristics are described in Table 1. Note that features such as gestational age duration (week), birth weight (g), birth order, mother's age at child's birth (year), mother's educational level, and family history of ALL (first degree relatives) did not differ significantly between the two groups (*p* > 0.05). The mean of patients' age at the time of diagnosis was 6.14 years (±4.33).

**Figure 1:**
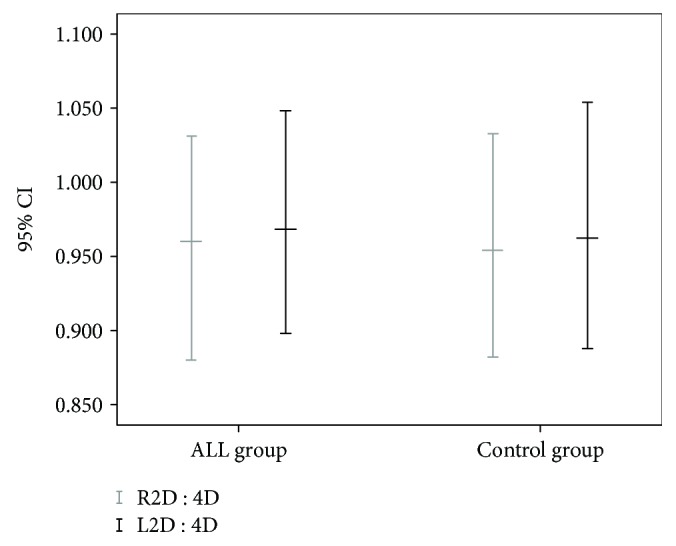
Confidence intervals of the 2D : 4D ratios of the right and left hands of patients with acute lymphoblastic leukemia (ALL group) and the control group.

Table 2 shows the means and standard deviations for the 2D : 4D ratios of the right and left hands and the differences between right-hand and left-hand 2D : 4D (DR-L) between the ALL and control groups for the whole sample as well as for the distribution by gender. No statistically significant difference was observed (*p* > 0.05). Note that the means and confidence intervals of the 2D : 4D ratios of the right and left hands for the ALL and control groups were very close, as shown in Figure 1.

## 4. Discussion

During the development of 2D : 4D, at least 19 skeletogenic genes are activated or deactivated by prenatal testosterone and estrogen [7]. The abnormal expression of homeobox gene *HOXA* is implicated in the development of acute leukemia in childhood [10]. This overexpression of *HOXA* seems to be associated with both pediatric acute myeloid leukemia and ALL. This gene is a critical regulator of normal blood cell development, and it is normally expressed very early in hematopoietic progenitors [9].

These observations strongly indicate that deregulation of *HOX* pathways is a dominant mechanism of leukemia transformation [10, 18]. Our working hypothesis was that intrauterine sex hormones (testosterone and progesterone) have been shown to regulate *HOX* gene expression, influencing the development of acute leukemia in childhood which could result in a specific pattern of 2D : 4D ratio in individuals affected by ALL since 2D : 4D has been proposed as a marker for prenatal hormone (testosterone and estrogen) exposure. However, the results obtained in this study did not support this hypothesis since the differences between the 2D : 4D ratios of both groups were not significant for any of the analyses performed.

When comparing the means of the 2D : 4D ratios of all participants between the two groups, we observed very similar averages. This suggests that prenatal estrogen levels in patients with ALL do not differ from those in clinically normal individuals, and that exposure to prenatal hormones does not appear to be associated with the occurrence of ALL.

Another result that confirms the absence of a specific digital standard for individuals with ALL was the presence of sexual dimorphism observed between the boys and girls in both groups. The mean for the 2D : 4D ratios of girls with ALL were greater than those of boys with the same condition. This result is supported by the literature, which states that in general men tend to have a shorter second digit than the fourth, and in women the second tends to be the same size or slightly longer than the fourth digit, leading to 2D : 4D ratios sexually dimorphic with mean male 2D : 4D ratios lower than mean female ratios [2].

Previous studies have provided mixed findings regarding an association of the 2D : 4D ratio with cancer [11, 18–20]. For instance, a study from Brazil with 100 patients diagnosed with prostate cancer showed a lower 2D : 4D ratio for affected than for healthy subjects [18]. Similarly, a study done in a group with 85 primary brain tumor patients reported 2D : 4D ratios lower relatively to healthy individuals suggesting greater prenatal testosterone and lower prenatal estrogen exposure in brain tumor patients [19]. On the other hand, higher 2D : 4D ratios were reported in patients with breast cancer and gastric cancer relative to age- and sex-matched controls [11, 20]. Other research did not find an association between 2D : 4D ratio and testicular cancer [21]. These mixed findings regarding an association of the 2D : 4D ratios with cancer suggest that prenatal sex steroid exposures can have different associations with different cancer types [19].

A critical review of the literature on direct versus indirect measurements of digit ratio recently points out that if a small number of participants are involved and there is adequate time to measure, then direct measurements are indicated [22]. We chose the direct technique for measuring the length of fingers using vernier calipers because of its significant reproducibility, low cost, and practicality.

Both 2D : 4D ratio and the prevalence of ALL show differences between races and ethnic groups. Black subjects have a lower digit ratio compared with white subjects, and contrastively white subjects present a higher incidence of ALL than black subjects [2]. Within this context, it is important to make some remarks about the Brazilian population's race. The Brazilian population was formed by an extensive mixture from three different ancestral roots: Amerindians, Europeans, and Africans. This resulted in a great variability of skin pigmentation, with no discontinuities between black and white skin colors [23]. Thus, the race of Brazilian individuals cannot be determined by skin color. The ideal method is to genetically identify the contribution of each ancestry to characterize the race of the study population. Several studies conducted in different Brazilian populations, including the population of the state of this present study, concluded that European ancestry is the major contributor to the genetic background of Brazilians [24, 25].

Due to the fact that ALL shows significant variability across geographic origin and racial and ethnic characteristics, as well as socioeconomic status, we suggest further studies in different populations, from different areas of Brazil with larger samples to fully uncover the relationship between 2D : 4D and ALL.

## 5. Conclusion

Based on the results obtained in this study, we observed that patients with ALL do not have a different digit pattern when compared with unaffected individuals, which may suggest that intrauterine exposure to sex hormones is similar between groups and that the 2D : 4D ratio may not be a marker for this specific type of cancer.

## Figures and Tables

**Table 1:** Characteristics of patients with acute lymphoblastic leukemia (ALL group) and control group.

Characteristics	ALL group *n* = 43 *n* (%)	Control group *n* = 86 *n* (%)	*p* value
*Gestational age* (*weeks*)			
<37	02 (4.7)	10 (11.6)	
37–41	38 (88.4)	70 (81.4)	0.435
≥42	03 (7.0)	06 (7.0)	
Mean (SD)	39.42 (2.805)	39.35 (2.170)	
*Birth weight* (*g*)			
<2500	04 (9.3)	11 (12.8)^∗^	
2500–3999	33 (76.7)	70 (81.4)^∗^	0.090
≥4000	06 (14)	03 (3.5)^∗^	
Mean (SD)	3.288 (0.626)	3.134 (0.626)	
*First born*			
Yes	14 (32.6)	36 (41.9)	0.307
No	29 (67.4)	50 (58.1)	
*Maternal age* (*years*)			
<35	37 (86.0)	79 (91.9)	0.301
≥35	6 (14.0)	7 (8.1)	
Mean (SD)	27.07 (6.720)	25.53 (5.895)	
*Maternal education* (*years*)			
<5	06 (13.9)	2 (2.3)	
5–8	11 (25.6)	23 (26.7)	0.38
≥9	26 (60.5)	61 (70.9)	
*Family history of ALL*			
Yes	0	0	
No	43	86	

Chi-squared test. ^∗^Missing.

**Table 2:** Comparison of right-hand digit ratio (R2D : 4D), left-hand digit ratio (L2D : 4D), and right-hand minus left-hand digit ratio (DR-L) among patients with acute lymphoblastic leukemia (ALL group) and the control group.

All participants, *n*	ALL group Mean (SD) 43	Control group Mean (SD) 86	*p* value	Cohen's *d*
R2D : 4D	0.9609 (0.0343)	0.9549 (0.0351)	0.292	0.172
L2D : 4D	0.9663 (0.0334)	0.9645 (0.0333)	0.767	0.054
DR-L	−0.0054 (0.0300)	−0.0104 (0.0259)	0.325	0.178
*Distribution by gender*				
*Girls, n*	**19**	**43**		
R2D : 4D	0.9675 (0.0309)	0.9651 (0.2757)	0.756	0.012
L2D : 4D	0.9723 (0.0308)	0.9701 (0.0314)	0.891	0.071
DR-L	−0.0037 (0.0262)	−0.0050 (0.0239)	0.852	0.052
*Boys, n*	**24**	**43**		
R2D : 4D	0.9557 (0.0366)	0.9434 (0.0390)	0.211	0.325
L2D : 4D	0.9624 (0.0355)	0.9598 (0.0349)	0.776	0.074
DR-L	−0.0067 (0.0332)	−0.0141 (0.0257)	0.243	0.249

Student's *t*-test for unpaired samples.

## Data Availability

The data used to support the findings of this study are available from the corresponding author upon request.
